# Association among biofilm formation, virulence gene expression, and antibiotic resistance in *Proteus mirabilis* isolates from diarrhetic animals in Northeast China

**DOI:** 10.1186/s12917-020-02372-w

**Published:** 2020-06-05

**Authors:** Yadong Sun, Shanshan Wen, Lili Zhao, Qiqi Xia, Yue Pan, Hanghang Liu, Chengwei Wei, Hongyan Chen, Junwei Ge, Hongbin Wang

**Affiliations:** 1 College of Veterinary Medicine, Northeast Agricultural University, Harbin, 150030 P.R. China; 2 Liaoning Vocational College of Ecological Engineering, Shenyang, 110122 P.R. China; 3 State Key Laboratory of Veterinary Biotechnology, Heilongjiang Provincial Key Laboratory of Laboratory Animal and Comparative Medicine, Harbin Veterinary Research Institute, Chinese Academy of Agricultural Sciences, Harbin, 150069 P.R. China; 4 Northeastern Science Inspection Station, China Ministry of Agriculture Key Laboratory of Animal Pathogen Biology, Harbin, 150030 P.R. China

**Keywords:** *Proteus mirabilis*, Animal, Diarrhea, Biofilm, Virulence gene, Antibiotic resistance, Pathogenicity

## Abstract

**Background:**

The aim of this study was to investigate the association among biofilm formation, virulence gene expression, and antibiotic resistance in *P. mirabilis* isolates collected from diarrhetic animals (*n* = 176) in northeast China between September 2014 and October 2016.

**Results:**

Approximately 92.05% of the isolates were biofilm producers, whereas 7.95% of the isolates were non-producers. The prevalence of virulence genes in the biofilm producer group was significantly higher than that in the non-producer group. Biofilm production was significantly associated with the expression of *ureC*, *zapA*, *rsmA*, *hmpA*, *mrpA*, *atfA*, and *pmfA* (*P* < 0.05). The results of drug susceptibility tests revealed that approximately 76.7% of the isolates were multidrug-resistant (MDR) and extensively drug-resistant (XDR). Biofilm production was significantly associated with resistance to doxycycline, tetracycline, sulfamethoxazole, kanamycin, and cephalothin (*P* < 0.05). Although the pathogenicity of the biofilm producers was stronger than that of the non-producers, the biofilm-forming ability of the isolates was not significantly associated with morbidity and mortality in mice (*P* > 0.05).

**Conclusion:**

Our findings suggested that a high level of multidrug resistance in *P. mirabilis* isolates obtained from diarrhetic animals in northeast China. The results of this study indicated that the positive rates of the genes expressed by biofilm-producing *P. mirabilis* isolates were significantly higher than those expressed by non-producing isolates.

## Background

*Proteus mirabilis* is a motile gram-negative bacillus belonging to the family *Enterobacteriaceae*. It is an opportunistic pathogen of great importance that is found in water and soil as well as in the intestinal tracts of mammals. It has been recognized as a leading cause of urinary tract infections [1] and the primary infectious factor in patients with indwelling urinary catheters [2]. *P. mirabilis* can cause food poisoning, respiratory and wound infections, bacteremia, and other infections [3–6]. In the past decade, diseases associating with *P. mirabilis* infection have also been reported in birds with reproductive failure [7], weaned infant rhesus monkeys (*Macaca mulatta*) and ferrets (*Mustela putorius furo*) with diarrhea [8], and dogs with chronic otitis externa [9]. Although *P. mirabilis*, as an opportunistic pathogen capable of causing serious infections, should not be neglected.

Various virulence factors contribute to the pathogenicity of *P. mirabilis*. These factors include the presence of fimbriae and specific outer membrane proteins; flagella-based and swarming motility; urease activity;environmental iron binding;cell invasiveness,in addition to lipopolysaccharides and hemolysins,most of which are involved in the ability of bacteria to adhere, colonize, and invade tissues, thereby promoting pathogenicity [10, 11]. Virulence gene expression is not the only factor responsible for the pathogenicity of *P. mirabilis*; biofilm formation exacerbates the complexity of *P. mirabilis* infection [12], as biofilms are recognized as the ultimate cause of persistent and destructive infections and inflammatory processes [13]. A biofilm is an assemblage of microbial cells that adhere to specific surfaces and neighboring cells, and it is covered with an extracellular matrix [14, 15]. Biofilms inadvertently contribute to bacterial survival, thereby enabling better adaptation to the conditions of the external environment and more effective use of nutrition [16]. *P. mirabilis* has been found to produce biofilms on a wide range of surfaces, including polystyrene, silicone, latex, glass, and various biological surfaces [17, 18]. In recent years, studies have reported a correlation between biofilm formation and various virulence factors in *P. mirabilis* isolates from humans [19, 20]. For instance, catheter encrustation has been reported to be brought about by the activity of urease-producing biofilms [19, 21]. Jansen et al. discovered that mannose-resistant *Proteus*-like fimbriae produced by *P. mirabilis*, which infects the urinary tract, can induce biofilm formation, with the fimbriae aiding in the aggregation of the bacteria [20]. However, most reports of *P. mirabilis* describe urinary tract infections in humans, and there are few reports on *P. mirabilis* isolated from diarrhetic animals. In addition, the association between biofilm formation and various virulence factors in *P. mirabilis* isolates from diarrhetic animals is still unknown.

Over the past two decades, due to the identification of multiple multidrug-resistant (MDR) and extensively drug-resistant (XDR) *P. mirabilis* isolates, the treatment of *P. mirabilis* infections has become increasingly difficult [22–25]. For instance, the production of biofilms by *P. mirabilis* exacerbates the complexity of bacterial resistance, prolongs the treatment time, and further aggravates the infection. In essence, biofilms protect organisms from the host immune system and antimicrobial agents [26]. Furthermore, a previous study has demonstrated that certain antibiotics can induce biofilm formation [27]. However, it is unclear whether antibiotics are linked to biofilm formation by *P. mirabilis* isolates obtained from diarrhetic animals.

Here, we investigate biofilm formation, antimicrobial susceptibility, and virulence gene expression in *P. mirabilis* isolates recovered from feces of various diarrhetic animals in northeast China and discuss the association among the pathogenicity, drug resistance, and virulence of *P. mirabilis* from the perspective of biofilm formation.

## Results

### Prevalence of *P. mirabilis* isolates

The prevalence rates of *P. mirabilis* in the diarrheal samples are listed in Table 1. Overall, 28.66% (176/614) of diarrheal specimens were positive for *P. mirabilis*. The positive isolation rate of *P. mirabilis* in different species was less than 40%. The positive rates of *P. mirabilis* isolated from specimens was 32.76% for dog, followed by 28.7% for mink, 23.26% for cattle, and 22.5% for fowl.

**Table 1:** Prevalence of *P. mirabilis* in collected diarrheal samples

Host	Number of samples	Number of positive samples (%)
Dog	232	76 (32.76)
Mink	216	62 (28.7)
Cattle	86	20 (23.26)
Fowl	80	18 (22.5)
Total	614	176 (28.66)

### Biofilm formation

Of the *P. mirabilis* isolates (*n* = 176) tested, 162 (92.05%) were biofilm producers and 14 (7.95%) were non-producers. Of the biofilm producers (*n* = 162), 78 (48.15%) were moderate biofilm producers, whereas 62 (38.27%) and 22 (13.58%) were strong and weak biofilm producers, respectively.

### Virulence gene expression

The prevalence rates of *ureC*, *zapA*, *rsmA*, *hmpA*, *mrpA*, *atfA*, *pmfA*, *FliL*, and *ucaA* in *P. mirabilis* are listed in Table 2. Of the *P. mirabilis* isolates, the most prevalent gene was *ureC*, which was identified in 90.91% of the isolates, followed by *zapA*, *rsmA*, *hmpA*, *mrpA*, *atfA*, *pmfA*, *FliL*, and *ucaA*, which were detected in 85.8, 81.25, 70.45, 65.91, 64.77, 60.23, 56.82, and 32.95% of the isolates, respectively. The positive rates of genes identified in the biofilm-producing isolates were significantly higher than those in the non-producing isolates. Furthermore, except for hmpA,eight genes tested in the moderate biofilm producing isolates were higher than those in the strong and weak biofilm producing isolates. All nine genes tested in this study showed the lowest prevalence rates among the non-producers. Biofilm production was significantly associated with the expression of *ureC*, *zapA*, *rsmA*, *hmpA*, *mrpA*, *atfA*, and *pmfA* (*P* < 0.05).

**Table 2:** Prevalence of *ureC, zapA, rsmA, hmpA, mrpA, atfA, pmfA, FliL* and *ucaA* genes in *P. mirabilis isolates*

Virulence gene	*P. mirabilis*				
	High biofilm producer *n* = 62	Moderate biofilm producer *n* = 78	Weak biofilm producer *n* = 22	Non biofilm producer *n* = 14	*P* value
*ureC*	58(93.54%)	76 (97.43%)	16 (72.72%)	10 (71.43%)	*P* < 0.0001
*zapA*	52 (83.87%)	72 (92.31%)	15 (68.18%)	12 (85.71%)	0.037
*rsmA*	50 (80.64%)	70 (89.74%)	14 (63.64%)	9 (64.29%)	0.013
*hmpA*	48 (77.42%)	60 (76.92%)	10 (45.45%)	6 (42.85%)	0.002
*mrpA*	41 (66.13%)	65 (83.33%)	8 (36.36%)	2 (14.29%)	*P* < 0.0001
*atfA*	40 (64.52%)	62 (79.49%)	10 (45.45%)	2 (14.29%)	*P* < 0.0001
*pmfA*	38 (61.29%)	58 (74.36%)	8 (36.36%)	2 (14.29%)	*P* < 0.0001
*FliL*	33 (53.23%)	50 (64.1%)	10 (45.45%)	7 (50%)	0.329
*ucaA*	14 (22.58%)	32 (41.03%)	7 (31.82%)	5 (35.71%)	0.146

### Antimicrobial susceptibility testing

**Fig. 1:**
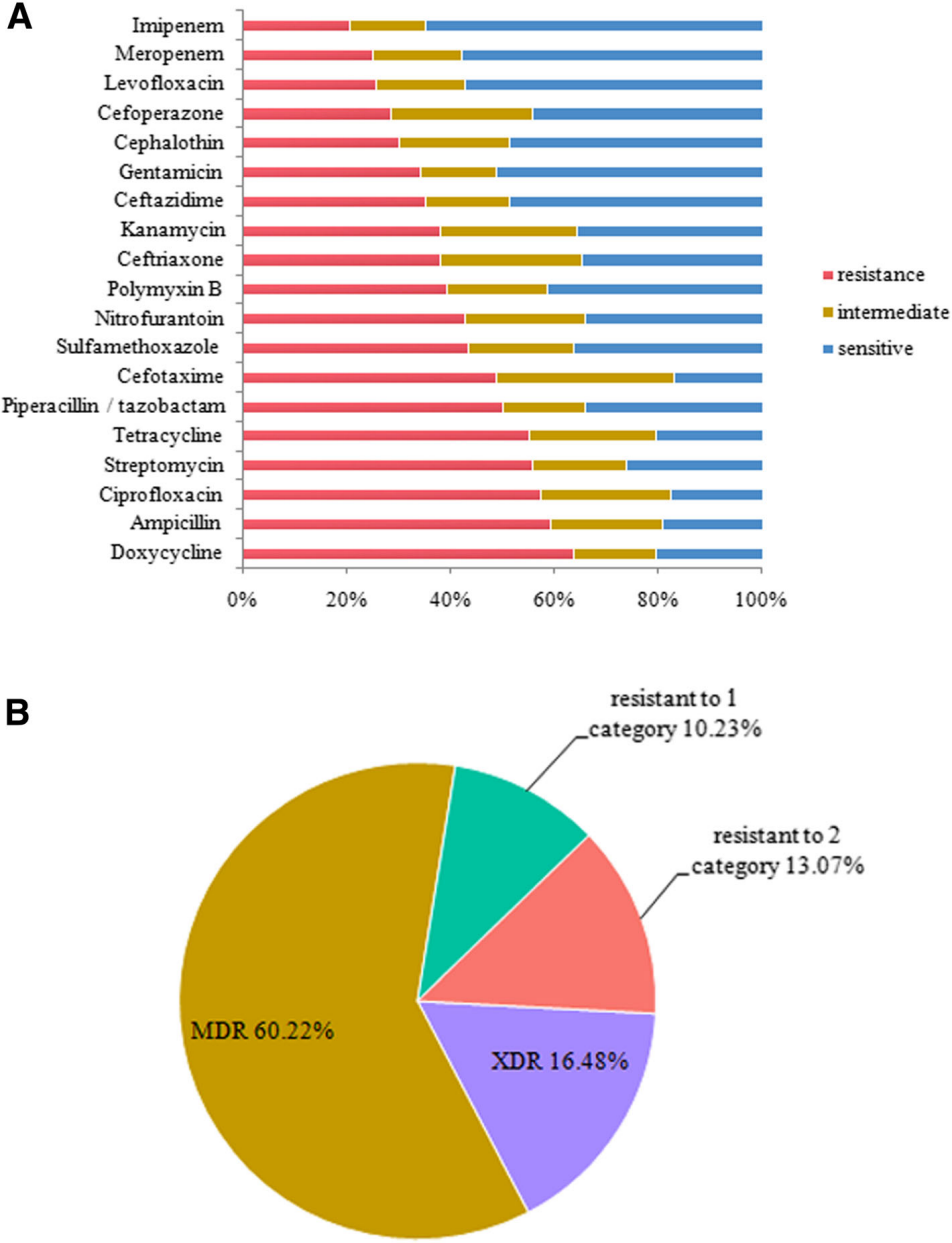
Antibiotic resistance phenotypes of *P. mirabilis* isolates examined in this study. **a** Resistance rates of all isolates to 19 antibiotics. **b** Approximately 76.7% of the isolates exhibited multidrug or extensive drug resistance

The antimicrobial resistance patterns of the *P. mirabilis* isolates are shown in Fig. 1. Variable degrees of resistance of these isolates to all antibiotics tested were observed. The resistance to doxycycline was the highest (112, 63.64%), followed by ampicillin (104, 59.09%), ciprofloxacin (101, 57.39%), streptomycin (98, 55.68%), tetracycline (97, 55.12%), piperacillin/tazobactam (88, 50%), cefotaxime (86, 48.87%), sulfamethoxazole (76, 43.19%), nitrofurantoin (75, 42.61%), polymyxin B (69, 39.2%), ceftriaxone (67, 38.07%), kanamycin (67, 38.07%), ceftazidime (62, 35.23%), gentamicin (60, 34.09%), cephalothin (53, 30.12%), cefoperazone (50, 28.41%), levofloxacin (45, 25.57%), meropenem (44, 25%), and imipenem (36, 20.45%). Of the sensitive strains, meropenem (57.96%) and imipenem (64.78%) showed the strongest antimicrobial effect on *P. mirabilis* (Fig. 1a).

The MDR patterns of the *P. mirabilis* isolates are shown in Fig. 1b. No isolate was sensitive to all the antibiotics. Of the *P. mirabilis* isolates, 18 and 23 isolates were resistant to only one or two of the 19 antibiotics tested, respectively, and 106 isolates were MDR, whereas 29 isolates were XDR. Thus, approximately 76.7% of the strains exhibited MDR or XDR.

The antibiotic susceptibility patterns of the biofilm producing and non-producing *P. mirabilis* isolates are shown in Table 3. Both biofilm producers and non-producers were highly resistant to doxycycline and moderately resistant to cefotaxime. Of the 19 antibiotics, doxycycline, ampicillin, tetracycline, cefotaxime, and kanamycin were found to be non-susceptible to non-producers. A sensitivity of 62.95% was observed for the biofilm-producing isolates to imipenem, whereas a sensitivity of 85.71% was noticed for the non-producing isolates to meropenem and imipenem. Isolates showing sensitivity to doxycycline, ampicillin, ciprofloxacin, streptomycin, tetracycline, piperacillin/tazobactam, cefotaxime, nitrofurantoin, sulfamethoxazole, and ceftriaxone produced more biofilm than strains showing resistance to these antibiotics. For other antibiotics, such as polymyxin B, ceftazidime, kanamycin, gentamicin, cefoperazone, cephalothin, meropenem, levofloxacin, and imipenem, we observed opposite findings. Biofilm production was significantly associated with resistance to doxycycline, tetracycline, sulfamethoxazole, kanamycin, and cephalothin (*P* < 0.05).

**Table 3:** Antibiotic resistance pattern of the biofilm producing and non-producing *P. mirabilis isolates*

	Biofilm producer (n = 162)			Non biofilm producer (n = 14)			
Antibiotic	resistance	intermediate	sensitive	resistance	intermediate	sensitive	*P* value
Doxycycline	98 (60.49%)	28 (17.28%)	36 (22.23%)	14 (100%)	0 (0%)	0 (0%)	0.015
Ampicillin	92 (56.79%)	36 (22.23%)	34 (20.98%)	12 (85.71%)	2 (14.29%)	0 (0%)	0.078
Ciprofloxacin	90 (55.56%)	44 (27.16%)	28 (17.28%)	11 (78.57%)	0 (0%)	3 (21.43%)	0.084
Streptomycin	88 (54.32%)	30 (18.52%)	44 (27.16%)	10 (71.42%)	2 (14.29%)	2 (14.29%)	0.445
Tetracycline	85 (52.47%)	41 (25.3%)	36 (22.23%)	12 (85.71%)	2 (14.29%)	0 (0%)	0.034
Piperacillin/tazobactam	84 (51.85%)	26 (16.05%)	52 (32.1%)	4 (28.58%)	2 (14.29%)	8 (57.13%)	0.15
Cefotaxime	78 (48.15%)	54 (33.33%)	30 (18.52%)	8 (57.13%)	6 (42.87%)	0 (0%)	0.255
Nitrofurantoin	67 (41.36%)	39 (24.07%)	56 (34.57%)	8 (57.13%)	2 (14.29%)	4 (28.58%)	0.492
sulfamethoxazole	64 (39.5%)	36 (22.23%)	62 (38.27%)	12 (85.71%)	0 (0%)	2 (14.29%)	0.004
Ceftriaxone	62 (38.27%)	45 (27.78%)	55 (33.95%)	5 (35.7%)	3 (21.43%)	6 (42.87%)	0.776
Polymyxin B	61 (37.66%)	32 (19.75%)	69 (42.59%)	8 (57.13%)	2 (14.29%)	4 (28.58%)	0.357
Ceftazidime	58 (35.8%)	28 (17.28%)	76 (46.92%)	4 (28.58%)	0 (0%)	10 (71.42%)	0.131
Kanamycin	57 (35.19%)	42 (25.93%)	63 (38.88%)	10 (71.42%)	4 (28.58%)	0 (0%)	0.005
Gentamicin	52 (32.1%)	24 (14.82%)	86 (53.08%)	8 (57.13%)	2 (14.29%)	4 (28.58%)	0.143
Cefoperazone	48 (29.63%)	46 (28.4%)	68 (41.97%)	2 (14.29%)	2 (14.29%)	10 (71.42%)	0.104
Cephalothin	45 (27.78%)	37 (22.84%)	80 (49.38%)	8 (57.13%)	0 (0%)	6 (42.87%)	0.030
Meropenem	42 (25.93%)	30 (18.52%)	90 (55.55%)	2 (14.29%)	0 (0%)	12 (85.71%)	0.061
Levofloxacin	39 (24.07%)	30 (18.52%)	93 (57.41%)	6 (42.87%)	0 (0%)	8 (57.13%)	0.117
Imipenem	36 (22.23%)	24 (14.82%)	102 (62.95%)	0 (0%)	2 (14.29%)	12 (85.71%)	0.114

### Pathogenicity test in mice

The morbidity and mortality of mice that received 32 different isolates of pathogenic bacteria is demonstrated in Table 4. Mice in the negative control group were obviously asymptomatic and in good overall health. Within 12 h of challenge, different symptoms of variable degrees were observed in mice of the experimental groups. These symptoms included gloomy spirit, inactivity, and loss of appetite. In the biofilm-producing group, some mice died within 12 h of challenge. Mice in both the biofilm producing and non-producing groups showed severe clinical symptoms, such as diarrhea, abrosia, subdued behavior, hunched appearance, and absence of grooming within 24 h of challenge. Eleven mice (45.83%) in the biofilm producing and non-producing groups died within 48 h of challenge. After 72 h of challenge, the symptoms began to gradually disappear, and the health of most mice returned to normal. Only two mice (25%) in the non-producing group died within 72 h of challenge. The biofilm-forming ability of *P. mirabilis* was not significantly associated with morbidity and mortality in mice (*P* > 0.05).

**Table 4:** Pathogenicity to mice in 32 biofilm producing and non-producing *P. mirabilis isolates* from animal with diarrhea

Pathogenicity	*P. mirabilis*		
	Biofilm producer (*n* = 24)	Non biofilm producer (*n* = 8)	*P* value
morbidity	17 (70.83%)	5 (62.5%)	0.660
mortality	11 (45.83%)	2 (25%)	0.299

## Discussion

Biofilm formation by *P. mirabilis* has recently become an issue of increasing concern. In a previous study, *P. mirabilis* isolates recovered from urine samples showed a higher degree of biofilm production than those isolated from different catheter segments [28]. In this study, the prevalence of *P. mirabilis* was high, which is in agreement with the results of studies on urine from catheterized patients [12, 28]. Taken collectively, these findings indicate that further studies on *P. mirabilis* biofilm formation are needed to better understand the disease process and to develop new preventive and therapeutic options.

As a target gene, *ureC*, was used to positively identify *P. mirabilis* as described previously [29]. Ali et al. reported that 96.66% of human *P. mirabilis* isolates (*n* = 30) recovered from the urinary tract expressed *ureC* [30]. In this study, 90.91% of the isolates were positive for *ureC*. However, its prevalence was relatively low when compared to the results of an earlier study [29]. Our findings revealed that testing only *ureC* increased the likelihood of obtaining *P. mirabilis* negative results. Therefore, we used a PCR method based on *16S rRNA* expression to detect *P. mirabilis*. The results of our study showed that the prevalence rates of *zapA*, *rsmA*, *hmpA*, *mrpA*, *atfA*, *pmfA*, *FliL*, and *ucaA* were relatively high. Furthermore, the overall prevalence of *P. mirabilis* was much higher in our study than that in previous studies [31–36].

The prevalence rates of eight genes expressed by moderate biofilm producers were significantly higher than those expressed by strong and weak biofilm producers. These results show that moderate biofilm producers are highly virulent. This is also the first study reporting that the biofilm-forming ability of *P. mirabilis* is significantly associated with the expression of *ureC*, *zapA*, *rsmA*, *hmpA*, *mrpA*, *atfA*, and *pmfA* (*P* < 0.05). Biofilm formation is associated with the adhesion and aggregation of bacteria [20], and *rsmA*, *mrpA*, and *atfA* have been reported to be involved in bacterial adhesion and aggregation [31–33], which is consistent with the results of this study. Biofilm formation was also associated with *ureC* expression, as previously reported [19, 21]. However, the association between *zapA* and *hmpA*, and biofilm formation needs further study.

The results of antimicrobial susceptibility tests revealed high resistance rates of the *P. mirabilis* isolates to several antibiotics such as doxycycline and ampicillin. The resistance rates ranged from 30 to 50%. The *P. mirabilis* isolates that were resistant to antibiotics were similar to those previously isolated from chicken products in Hong Kong [37]. However, the resistance rates of *P. mirabilis* to streptomycin, sulfamethoxazole, kanamycin, ampicillin, ciprofloxacin, cephalothin, gentamicin, cefotaxime, and ceftazidime were significantly higher than those reported in an earlier study, which isolated *P. mirabilis* from dogs [38]. Our results on the resistance rates of *P. mirabilis* to nitrofurantoin, tetracycline, and polymyxin B were much lower than those reported in a human study [39], although the resistant rates to other antibiotics, except nitrofurantoin, tetracycline, and polymyxin B, were generally high [39, 40]. In a previous study, *P. mirabilis* isolates from dogs were found to be highly sensitive to ciprofloxacin and gentamicin [41]. In this study, meropenem and imipenem were the most effective antibiotics against *P. mirabilis*, which is consistent with the results of an earlier study [42]. Meropenem and imipenem are classified as carbapenems, and our results showed that carbapenems were highly effective against *P. mirabilis*. We also found that approximately 76.7% of the isolates were MDR or XDR, which is very high compared to the rate reported in a previous study [43]. This finding reveals that resistance to *P. mirabilis* was steadily increasing. It is also important to mention that the high number of MDR *P. mirabilis* isolates obtained from companion animals may pose a potential threat to human health. Further studies are needed to define the mechanism of resistance, which may improve the treatment of *P. mirabilis* infections in the future.

In this study, we found that the resistance to several antibiotics, including piperacillin/tazobactam, ceftazidime, cefoperazone, ceftriaxone, meropenem, and imipenem, was significantly higher among biofilm producers than non-producers, indicating that biofilm producers were more resistant to antibiotics than non-producers. Presently, few studies have described an association between biofilm formation and drug resistance of *P. mirabilis*, although similar studies have been performed for other pathogens such as uropathogenic *E. coli* [44, 45], coagulase-negative staphylococci [46], and *Haemophilus parasuis* [47]. Except for piperacillin/tazobactam, ceftazidime, cefoperazone, ceftriaxone, meropenem, and imipenem, the antibiotic resistance rates of non-producers were higher than those of producers. This finding is consistent with an earlier study that reported non-MDR *Acinetobacter baumannii* isolates to participate in robust biofilm formation [27]. Different resistance mechanisms are likely to be responsible for the differences in antibiotic resistance and biofilm formation in various bacteria. We also found that the biofilm-forming ability of the *P. mirabilis* isolates was significantly associated with the resistance to doxycycline, tetracycline, sulfamethoxazole, kanamycin, and cephalothin (*P* < 0.05). β-lactamase has been reported to decrease the ability of *E. coli* to form biofilms by inhibiting peptidoglycans, which are required for the assembly of surface molecules on the biofilm. Under sub-inhibitory concentrations of tetracycline and ampicillin, the overexpression of the TetA(C) pump, which contributes to the osmotic stress response and induces capsular colanic acid production, promoted the formation of mature biofilms [48]. In addition, there was an association between the resistance to aminoglycoside and extracellular DNA in *Pseudomonas aeruginosa* [49]. Thus, we hypothesize that antibiotic resistance is associated with the composition of *P. mirabilis* biofilms, thereby affecting biofilm formation.

Although the pathogenicity of the biofilm producers was stronger than that of the non-producers, the biofilm-forming ability of the isolates was not significantly associated with morbidity and mortality in mice (*P* > 0.05). The high pathogenicity of the biofilm producers may have been due to the fact that the biofilm-forming ability of *P. mirabilis* associated with the expression of *ureC*, *zapA*, *rsmA*, *hmpA*, *mrpA*, *atfA*, and *pmfA* (*P* < 0.05). Furthermore, it has been reported that *ureC* and *zapA* expressed by *P. mirabilis* associate with diarrhea in goats [50]. Gabidullin et al. found that diarrhea caused by *P. mirabilis* was related to enterotoxins [51]. Thus, the mechanism responsible for *P. mirabilis*-induced diarrhea needs further study.

## Conclusions

In conclusion, our study revealed a high level of multidrug resistance in *P. mirabilis* isolates recovered from diarrhetic animals in northeast China. Therefore, the use of antimicrobial agents in animals needs to be controlled so as to minimize the emergence and eventual spread of resistant pathogens, which is warranted in order to protect human health. The results of this study indicated that the positive rates of the genes expressed by biofilm-producing *P. mirabilis* isolates were significantly higher than those expressed by non-producing isolates. Furthermore, this is the first study to report that the biofilm-forming ability of *P. mirabilis* isolates from diarrhetic animals is significantly associated with the expression of *ureC*, *zapA*, *rsmA*, *hmpA*, *mrpA*, *atfA*, and *pmfA* and the resistance to doxycycline, tetracycline, sulfamethoxazole, kanamycin, and cephalothin. Further studies on biofilm formation by *P. mirabilis* are necessary to better understand the disease process and to develop effective treatments for mammals with antibiotic resistant *P. mirabilis*.

## Methods

### Sample collection

Six hundred and fourteen fecal swabs were collected randomly from different farms in northeast China between September 2014 and October 2016 and transferred to the laboratory in ice-filled containers. Fecal swabs were collected from different animals (i.e., dog, mink, cattle, and fowl) with diarrhea.

### *P. mirabilis* screening

*P. mirabilis* was isolated as described previously [37]. To grow the bacteria, the fecal swabs were incubated with 1 mL of Luria–Bertani broth for 6 h at 37 °C, and a 30 μL aliquot of the broth was added to buffered peptone water (Aobox, Beijing, China). The resultant mixture was incubated for 24 h at 37 °C to enrich the culture, and then streaked onto xylose lysine deoxycholate agar plates (Hopebiol, Qingdao, China). All plates were incubated at 37 °C for a minimum of 16 h. One isolate from each sample was selected for further characterization.

### Identification of isolates

To identify *P. mirabilis* isolates, standard biochemical tests were used as described previously [52]. The isolates were gram-negative bacilli that were positive for glucose, methyl red, and urease but negative for maltose, sucrose, mannitol, indole, and gelatin liquefaction. The Voges–Proskauer test yielded negative results for the isolates. Urease C (*ureC*) and mannose-resistant *Proteus*-like fimbriae (*mrpA*) were amplified by the polymerase chain reaction (PCR) to identify *P. mirabilis* from the presumptive isolates [36]. Positive results were randomly selected for sequencing. When the results were negative for *ureC* and *mrpA* expression, a segment of the *16S rRNA* gene was amplified. The *16S rRNA* segment was sequenced [53]. The sequences were compared with *ureC*, *mrpA*, and *16S rRNA* in GenBank using BLAST.

### Quantification of biofilm production

Biofilm production was assessed using a microtiter plate assay as described previously [54]. *P. mirabilis* isolates were grown in tryptic soy broth (Hopebiol) supplemented with 0.25% glucose overnight at 37 °C. Subsequently, the cultures were diluted to 1 × 10^6^ CFU mL^− 1^ in fresh tryptic soy broth supplemented with 0.25% glucose, and 200 μL of the diluted culture was transferred into each well of a sterile flat-bottom 96-well polystyrene microtiter plate. After incubation for 24 h at 37 °C without shaking, the wells were gently washed three times with 200 μL of distilled water. The biofilms were fixed with 200 μL of 99% methanol for 15 min. The supernatant was removed, and the plate was air-dried. The biofilms were then stained with 200 μL of 1% crystal violet for 10 min. The excess dye was removed by washing the plate under running tap water. Finally, the bound crystal violet was released by adding 200 μL of 33% acetic acid. The optical density (OD) of each well was measured at 590 nm using a microtiter plate reader. To determine the background OD, control experiments were performed with uninoculated broth. This assay was repeated three independent times. The biofilm formation capacity of each isolate was analyzed using the method of Khoramian et al. [55].

### Expression of virulence genes

*P. mirabilis* virulence genes were detected by PCR. These genes were *ureC*, extracellular metalloprotease (*zapA*), swarming behavior (*rsmA*), hemolysin (*hpmA*), *mrpA*, ambient-temperature fimbriae (*atfA*), *P. mirabilis* fimbriae (*pmfA*), flagellar basal body protein (*FliL*), and uroepithelial cell adhesin fimbriae (*ucaA*) (Table 5) [10]. The base sequences and predicted sizes of the PCR products for the specific oligonucleotide primers used in this study are listed in Table 5. Subsequently, total genomic DNA was isolated from stationary-phase broth cultures that were grown overnight in Luria–Bertani broth with the TIANamp Bacterial DNA Kit (TIANGEN, Beijing, China) according to the manufacturer’s instructions. All PCR products were analyzed by agarose gel electrophoresis, and the results were recorded with a gel documentation system. All amplifications were repeated three independent times in parallel with a negative control (distilled water served as the PCR template).

**Table 5:** Primers used in the PCRs carried out in this study

Target gene	Primer	Nucleotide Sequence (5′-3′)	Amplicon (bp)	AT*	Reference
*16S rRNA*	*27*F	AGAGTTTGATCCTGGCTCAG	1463	49	Leite et al. (2015) [53]
	*1492*R	GGTTACCTTGTTACGACTT			
*ureC*	*ureC*-F	GTTATTCGTGATGGTATGGG	317	52	Stankowska et al. (2008) [36]
	*ureC*-R	ATAAAGGTGGTTACGCCAGA			
*zapA*	*zapA*-F	ACCGCAGGAAAACATATAGCCC	540	53	Stankowska et al. (2008) [36]
	*zapA*-R	GCGACTATCTTCCGCATAATCA			
*rsmA*	*rsmA*-F	TAGCGAGTGTTGACGAGTGG	562	49	Shi et al. (2016) [31]
	*rsmA*-R	AGCGAGGTGAAGAACGAGAA			
*hpmA*	*hpmA*-F	CCAGTGAATTAACGGCAGGT	654	49	Shi et al. (2016) [31]
	*hpmA*-R	CGTGCCCAGTAATGGCTAAT			
*mrpA*	*MRP*-F	ACACCTGCCCATATGGAAGATACTGGTACA	550	40	Barbour et al. (2012) [32]
	*MRP*-R	AAGTGATGAAGCTTAGTGATGGTGATGGTG			
		ATGAGAGTAAGTCACC			
*FliL*	*FliL*-F	CTCTGCTCGTGGTGGTGTCG	770	40	Barbour et al. (2012) [32]
	*FliL*-R	GCGTCGTCACCTGATGTGTC			
*ucaA*	*ucaA*-F	GTAAAGTTGTTGCGCAAAC	560	50	Sosa et al. (2006) [35]
	*ucaA*-L	TTGAGCCACTGTGGATACA			
*pmfA*	*pmfA*-F	CAAATTAATCTAGAACCACTC	618	54	Zunino et al. (2003) [34]
	*pmfA*-R	ATTATAGAGGATCCCTTGAAGGTA			
*atfA*	*atfA*-F	CATAATTTCTAGACCTGCCCTAGCA	382	50	Zunino et al. (2000) [33]
	*atfA*-R	CTGCTTGGATCCGTAATTTTTAACG			

AT* anneling temperature

### Antimicrobial resistance test

The antimicrobial resistance of the isolates to 19 antibiotics, including doxycycline, ampicillin, ciprofloxacin, streptomycin, tetracycline, piperacillin/tazobactam, cefotaxime, nitrofurantoin, sulfamethoxazole, ceftriaxone, polymyxin B, ceftazidime, kanamycin, gentamicin, cefoperazon, cephalothin, meropenem, levofloxacin, and imipenem (BIO-KONT, Wenzhou, China), was determined by the Kirby–Bauer disc diffusion method [56]. In brief, 0.5 McFarland *P. mirabilis* inoculum was spread onto Mueller–Hinton agar plates (Hopebiol), and the antibiotic discs were dispensed on the agar. The plates were incubated at 37 °C for 18 h. *Escherichia coli* (*E. coli*) ATCC 25922 was used as the control microorganism. The inhibitory zone around each disc was measured, and the results were interpreted according to the guidelines provided by the manufacturers and the Clinical and Laboratory Standards Institute [57]. The results were interpreted as resistant, intermediate, and susceptible. MDR was defined as acquired non-susceptibility to at least one agent in three or more antimicrobial categories. XDR was defined as non-susceptibility to at least one agent in all but two or fewer antimicrobial categories [58]. Non-MDR was defined as resistance to none or up to two antimicrobial categories [27].

### Pathogenicity test in mice

The pathogenicity test was performed as described previously [59]. In brief, healthy, 6-week-old, female BALB/c mice (*n* = 99) (Changsheng Biotechnology Co., Ltd., Liaoning, China) were used to investigate the pathogenicity of *P. mirabilis*. The mice were randomly divided into thirty-three groups (*n* = 3/per group) as follows: one control group and thirty-two experimental groups, which tested 32 *P. mirabilis* isolates for their pathogenicity to produce biofilm, to express various virulence genes, and to resist different drugs. Each mouse received a 0.2-mL intraperitoneal injection of a bacterial suspension at a concentration of 1 × 10^8^ CFU mL^− 1^, and the control mice were challenged with vehicle alone. All the mice were fed normally and observed daily for the activity level and water intake for up to 14 days, and deaths were recorded,then the mice alive were euthanized by intraperitoneal injection of 40 mg/kg b.w. of sodium thiopental [60].

### Statistical analysis

Data were analyzed with SPSS 18.0 Software (SPSS Statistics, Inc., Chicago, IL, USA). The Chi-square test was adopted for analysis. *P*-values < 0.05 were considered statistically significant.

## Data Availability

The datasets generated and/or analysed during the current study are available in National Center for Biotechnology Information (NCBI) repository, under these GenBank accession numbers MT294143-MT294148 and MT276297-MT276312.

## Abbreviations

*P. mirabilis*: *Proteus mirabilis*
PCR: Polymerase chain reaction
MDR: Multidrug-resistant
XDR: Extensively drug-resistant
BPW: Buffered peptone water
OD: Optical density
*E. coli*: *Escherichia coli*
AT: Annealing temperature
